# Image-encoded biological and non-biological variables may be used as shortcuts in deep learning models trained on multisite neuroimaging data

**DOI:** 10.1093/jamia/ocad171

**Published:** 2023-09-05

**Authors:** Raissa Souza, Matthias Wilms, Milton Camacho, G Bruce Pike, Richard Camicioli, Oury Monchi, Nils D Forkert

**Affiliations:** Department of Radiology, Cumming School of Medicine, University of Calgary, Calgary, AB T2N 4N1, Canada; Hotchkiss Brain Institute, University of Calgary, Calgary, AB T2N 4N1, Canada; Biomedical Engineering Graduate Program, University of Calgary, Calgary, AB T2N 4N1, Canada; Hotchkiss Brain Institute, University of Calgary, Calgary, AB T2N 4N1, Canada; Alberta Children’s Hospital Research Institute, University of Calgary, Calgary, AB T2N 4N1, Canada; Department of Pediatrics, University of Calgary, Calgary, AB T2N 4N1, Canada; Department of Community Health Sciences, University of Calgary, Calgary, AB T2N 4N1, Canada; Department of Radiology, Cumming School of Medicine, University of Calgary, Calgary, AB T2N 4N1, Canada; Hotchkiss Brain Institute, University of Calgary, Calgary, AB T2N 4N1, Canada; Biomedical Engineering Graduate Program, University of Calgary, Calgary, AB T2N 4N1, Canada; Department of Radiology, Cumming School of Medicine, University of Calgary, Calgary, AB T2N 4N1, Canada; Hotchkiss Brain Institute, University of Calgary, Calgary, AB T2N 4N1, Canada; Department of Medicine (Neurology), Neuroscience and Mental Health Institute, University of Alberta, Edmonton, AB T6G 2E1, Canada; Hotchkiss Brain Institute, University of Calgary, Calgary, AB T2N 4N1, Canada; Department of Radiology, Radio-Oncology and Nuclear Medicine, Université de Montréal, Montréal, QC H3C 3J7, Canada; Centre de Recherche, Institut Universitaire de Gériatrie de Montréal, Montréal, QC H3W 1W4, Canada; Department of Clinical Neurosciences, Cumming School of Medicine, University of Calgary, Calgary, AB T2N 4N1, Canada; Department of Radiology, Cumming School of Medicine, University of Calgary, Calgary, AB T2N 4N1, Canada; Hotchkiss Brain Institute, University of Calgary, Calgary, AB T2N 4N1, Canada; Alberta Children’s Hospital Research Institute, University of Calgary, Calgary, AB T2N 4N1, Canada; Department of Clinical Neurosciences, Cumming School of Medicine, University of Calgary, Calgary, AB T2N 4N1, Canada

**Keywords:** shortcut learning, deep learning, multisite classification, MRI, data harmonization

## Abstract

**Objective:**

This work investigates if deep learning (DL) models can classify originating site locations directly from magnetic resonance imaging (MRI) scans with and without correction for intensity differences.

**Material and Methods:**

A large database of 1880 T1-weighted MRI scans collected across 41 sites originally for Parkinson’s disease (PD) classification was used to classify sites in this study. Forty-six percent of the datasets are from PD patients, while 54% are from healthy participants. After preprocessing the T1-weighted scans, 2 additional data types were generated: intensity-harmonized T1-weighted scans and log-Jacobian deformation maps resulting from nonlinear atlas registration. Corresponding DL models were trained to classify sites for each data type. Additionally, logistic regression models were used to investigate the contribution of biological (age, sex, disease status) and non-biological (scanner type) variables to the models’ decision.

**Results:**

A comparison of the 3 different types of data revealed that DL models trained using T1-weighted and intensity-harmonized T1-weighted scans can classify sites with an accuracy of 85%, while the model using log-Jacobian deformation maps achieved a site classification accuracy of 54%. Disease status and scanner type were found to be significant confounders.

**Discussion:**

Our results demonstrate that MRI scans encode relevant site-specific information that models could use as shortcuts that cannot be removed using simple intensity harmonization methods.

**Conclusion:**

The ability of DL models to exploit site-specific biases as shortcuts raises concerns about their reliability, generalization, and deployability in clinical settings.

## Introduction

Research has shown that machine learning, especially deep learning (DL) models, can achieve accuracies similar to human experts in many domains, including healthcare.^1^^,^^2^ These data-driven advancements in precision medicine may reduce healthcare costs by diagnosing diseases earlier and better, preventing diseases before becoming clinically evident, and providing better patient-specific treatment.^3^ To date, machine learning techniques have been successfully applied in several healthcare specialties, such as radiology^4^ and cardiology.^5^

Convolutional neural networks (CNNs), a DL method specifically designed for image analysis,^1^^,^^3^ have great potential to support many clinically relevant prediction, classification, and segmentation tasks using medical images.^6^^,^^7^ However, in order for CNNs to achieve high accuracy and precision, a large number of diverse training samples is typically required to capture the full real-world variability of the problem at hand.^1^ The standard approach to increasing the number and diversity of training samples in healthcare is to collect multisite data into a central database before training.

Generally, the training data need to represent the population of interest well in order to train a machine learning model that generalizes well to new institutions. If the data used during training differ from the real world, the corresponding models will likely achieve low performance when tested on new data. This may lead to the model performing poorly in real-world scenarios and may also introduce considerable biases in the model.

Within this context, several researchers have shown that biological and/or non-biological biases can influence DL models as well as traditional statistical analyses, such as voxel-based morphometry, for single-site and multisite applications using magnetic resonance imaging (MRI) data.^8–13^ Briefly described, biases can be categorized into biological (participants’ cohorts) and non-biological (technical) sources. Biological biases include variables such as sex, age, and other demographic variables, while non-biological biases involve differences regarding the number and class distribution of participants per site, imaging acquisition protocols, and scanners. While it is generally accepted in the MRI domain that the hardware and the protocols used can lead to significant differences in the resulting images, it remains unclear how much of an effect this has on DL models, especially in case of large multicenter studies. Practically, these differences can manifest as local and global distortions, imaging artifacts (eg, eddy currents), and variations in intensity distributions of non-quantitative imaging data resulting from differences in acquisition parameters and scanner hardware. This becomes particularly relevant when combining data collected acquired using non-harmonized imaging protocols from multiple sites to increase data diversity and size for machine learning training. Machine learning models trained using such data may exploit these differences as shortcuts, learning features associated with the specific site and its corresponding patient distribution, rather than relevant imaging patterns for the intended clinical task. Consequently, deploying such models in clinical centers that did not contribute to the training data collection may pose significant challenges, as the model may not rely on disease-related patterns for accurate predictions but false shortcuts that do not apply to the new data.

Geirhos et al.^14^ identified 4 shortcut opportunities, briefly described in the following. First, models can identify features that are different from the ones intended by model developers to accomplish their tasks, for example, the scanner type. Second, they can combine various features to inform their decision, for instance, disease status and scanner types. Third, models usually operate with minimum effort. More precisely, once a shortcut is found, the model can rely entirely on it, even though it represents an artifact of the data. Finally, models can focus on the majority group while accepting misclassifications for the minority group(s).

To overcome this potential problem, data harmonization techniques are often applied in practice. For example, rather simple linear intensity normalization methods such as calculating the mean and standard deviation of the image intensities for normalization (z-score normalization) or normalizing the image intensities with respect to a reference image (histogram matching) are often used as a preprocessing step of the image data prior to training of DL models. However, it remains unclear if rather simple intensity harmonization techniques are really suitable for removing the relevant biases that could be used for shortcut learning.

Therefore, the aim of this work was to develop a site classifier based on a large clinical database consisting of T1-weighted MRI brain scans originally collected for the classification of patients with Parkinson’s disease (PD) and investigate the effect of simple intensity harmonization techniques on the ability of the machine learning model to classify the originating sites. The created database covers all aspects of data heterogeneity (biological and non-biological) expected when working with multisite datasets. Therefore, the major contributions of this work can be summarized as follows: (1) the implementation of a site classifier using neuroimaging data from 41 different sites, and (2) the first evaluation of the effect of 3 biological variables (sex, age, and disease status) and one non-biological variable (scanner type) on the accuracy of the model using the raw as well as harmonized data.

## Materials and methods

### Dataset

**Figure 1.**
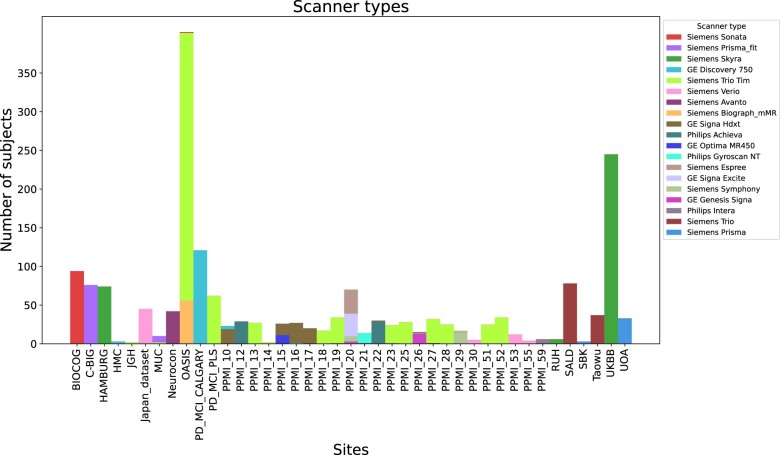
Scanner type distribution per site.

A multisite database was created by collecting datasets from patients with PD and healthy participants acquired across 41 different sites managed by 12 studies.^15–26^ In total, 1880 high-resolution T1-weighted brain MRI scans were included in this work. Table 1 summarizes the database distribution with respect to biological variables (refer to Table S1 for site-specific information). A variety of scanners and protocols were used during imaging acquisition. Siemens, GE, and Phillips were among the manufacturers with magnetic field strengths of either 1.5 or 3.0 T. Figure 1 shows the scanner type distribution per site (refer to Figures S1–S3 for sex, age, and disease status distributions).

**Table 1.** Sex and age distributions per subject characteristic.

Characteristic	Males	Females	Young (<60)	Old (60+)
Parkinson’s disease	542	325	222	645
Healthy participants	635	378	243	770

Each study received ethics approval from their local ethics board and received written informed consent from all the participants in accordance with the declaration of Helsinki.

### Dataset preprocessing

**Figure 2.**
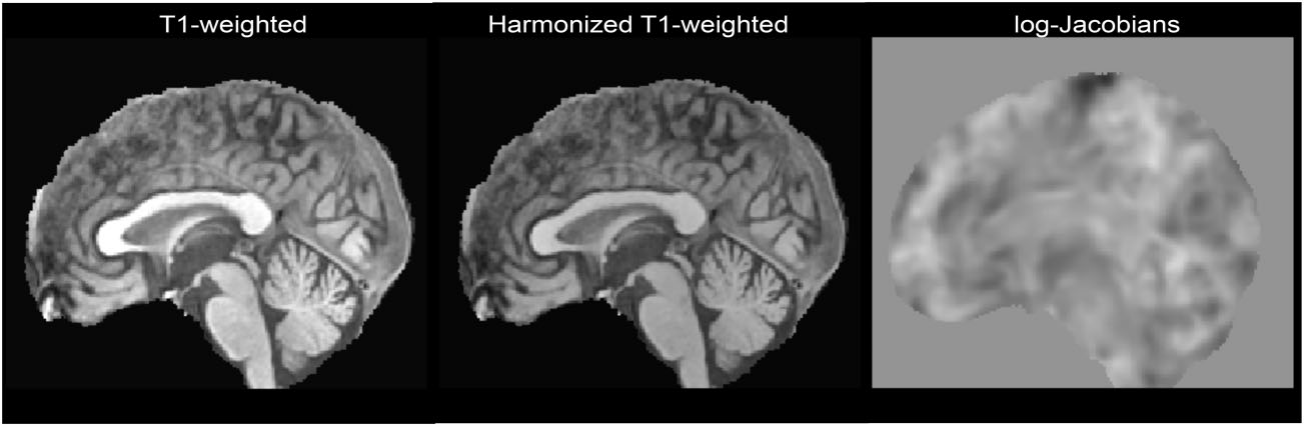
Example imaging data. T1-weighted denotes the preprocessed raw MRI brain scans, harmonized T1-weighted denotes the T1-weighted MRI scans after intensity harmonization (histogram matching), and log-Jacobians denotes the deformation fields generated during registration.

Three different imaging data types were used to train DL models to classify originating sites (see Figure 2): raw T1-weighted MRI scans, harmonized T1-weighted MRI scans, and log-Jacobians maps. A detailed explanation of how each image type was generated is provided in the following.

The collected database was preprocessed as follows. First, skull stripping was performed using HD-BET.^27^ Second, the scans were resampled to an isotropic resolution of 1 mm using linear interpolation. Third, bias field correction was applied using the Advanced Normalization Tools (ANTs) nonparametric nonuniform intensity normalization technique (version 2.3.1).^28^ After that, each scan was registered to the PD25-T1-MPRAGE-1mm brain atlas^29^ from the Montreal Neurological Institute (MNI) (fixed image) using ANTs. Registration of the scans to the atlas was performed in 2 steps. First, ANTs were used to affinely align the scans to the atlas. These scans were used as the raw T1-weighted MRI data type after cropping them to 160 × 192 × 160 voxels to eliminate extraneous background voxels and decrease computational strain during site classifier training as described in the next sections. Next, the affine registration was used to initialize a nonlinear registration step. The displacement fields resulting from the nonlinear registration were then utilized to generate the associated log-Jacobian maps^30^ as a means of data harmonization. Briefly described, log-Jacobians are symmetric around zero and indicate local changes in volume between the atlas and the individual scans for each voxel. Thus, values less than zero reflect a loss in volume. In contrast, values greater than zero represent an increase in volume. It can be assumed that any difference in intensity distribution should be removed from these log-Jacobian maps and that they are mainly representing morphological characteristics of the brain analyzed. The MNI PD25 brain mask was then used to crop the log-Jacobian maps to remove information outside the brain (background voxels).

After the preprocessing steps, which generated the raw T1-weighted MRI scans and log-Jacobian maps, intensity-harmonized T1-weighted scans were generated by applying histogram matching,^31^ an intensity harmonization technique. This technique normalizes the intensity values of each raw T1-weighted MRI scan based on a reference scan to account for site differences.^13^^,^^32^ In this work, the brain atlas used for registration was used as a reference for intensity harmonization.

Thus, it is expected that each image type will present different levels of site-specific information. (1) Raw T1-weighted scans likely contain the most discriminatory information to perform site classification, (2) harmonized T1-weighted scans may remove some site information encoded in the grey values, and (3) log-Jacobian maps should eliminate most site information as they contain mainly morphological information.

### Deep learning model

This work uses the state-of-the-art simple fully convolutional network (SFCN)^33^ as the basis for the site classifier because it achieved high performance for adult brain age prediction and sex classification using T1-weighted MRI datasets.^6^^,^^8^ Our DL architecture consisted of 7 blocks: The first 6 blocks are identical to the original SFCN model, with 5 blocks containing a 3D convolutional layer with a 3 × 3 × 3 kernel, batch normalization, 2 × 2 × 2 max pooling, and ReLU activation, and one block including a 3D convolutional layer with a 1 × 1 × 1 kernel, batch normalization, and ReLU activation. The final block was adapted for our task and consisted of a 3D average pooling layer, a dropout layer with a rate of 0.2, a flattening layer, and a multiclass classification layer with softmax activation to classify the originating site. The created database was stratified based on the number of subjects available at each site. More precisely, 80% of the MRI scans provided by each site were randomly selected for training and 20% for the testing set to develop and evaluate the originating acquisition site classifier. The Adam optimizer, with an initial learning rate of 0.001 and a decay rate of 0.003, was utilized for training the networks. Early stopping with patience of 10 was applied, and the best models (lowest validation loss) were saved for evaluation.

### Simulated data distribution

As a baseline for our work, we generated ten artificial sites by randomly splitting the created database, assigning 188 MRI scans to each artificial site, 148 scans for training, and 40 for testing. This way, every site has a more similar distribution (less biased), which should result in lower site classification accuracies when compared to the models trained in the real data distribution, for which models may learn site-dependent information as shortcuts.^14^

### Metrics and evaluation

In this study, a total of 6 site classifiers were trained and evaluated using the centrally collected scans from all centers. Among them, 3 were trained to analyze the real data distribution, while the remaining 3 were applied to evaluate the simulated data distribution. Each classifier utilized distinct types of imaging data as input, including raw T1-weighted MRI scans, intensity-harmonized T1-weighted MRI scans, or log-Jacobians maps.

All models were evaluated with respect to their ability to correctly classify the originating sites (accuracy). Furthermore, site classifiers classification rates based on sex, age, disease status, and scanner types were computed. In this context, classification rate refers to the percentage of subjects that had their sites correctly identified when grouped by each variable.

Additionally, a statistical likelihood ratio test analysis of the contribution of each variable was performed using logistic regression. In this case, multiple regression models were computed by dropping one of the variables of interest per time. Then, a likelihood ratio test was conducted between the models with dropped variables and a base model, which includes all variables, to determine how significant the effect of each variable is to the trained model.

Lastly, saliency maps were generated to display the brain regions contributing the most to the models’ decisions when classifying originating sites. In this work, the SmoothGrad^34^ method available in the tf-keras-vis toolkit^35^ was employed using the trained models, and up to 5 (in case fewer participants were available, all of them were included) correctly classified participants from each site from the testing set. In this method, Gaussian noise is added to the data before computing the standard saliency map, and the resulting maps are averaged. The saliency maps were averaged over 20 noisy samples per participant and the noise was sampled from a zero-mean Gaussian distribution with 0.2 variances.

## Results

**Figure 3.**
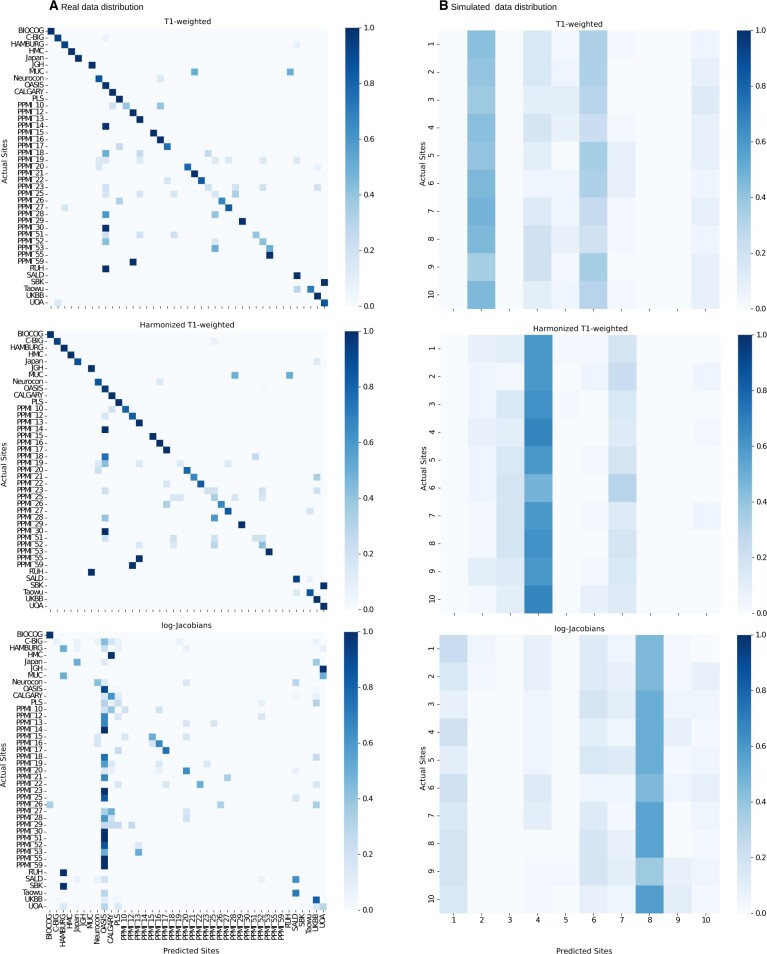
Site classification rates in a confusion matrix. (A) illustrates the results of the site classifiers using real data distribution while (B) presents the results using simulated data distribution.

Table 2 summarizes accuracy and classification rates grouped by sex, age, disease status, and scanner types for the models trained on the real data distribution, while Figure 3 shows site-specific performance in a confusion matrix for real and simulated data distributions.

**Table 2.** Site classifier accuracy and classification rates stratified by biological and non-biological variables per input type.

Site classifier performance		Site classifier classification rates grouped by			
Input	Accuracy	Sex (M/F)	Age (<60/60+)	Disease status (PD/HP)	Averaged scanner types
T1-weighted	0.85	0.82/0.90	0.81/0.86	0.75/0.93	0.88
Harmonized T1-weighted	0.85	0.83/0.88	0.80/0.87	0.77/0.92	0.86
Log-Jacobians	0.54	0.56/0.52	0.36/0.60	0.32/0.74	0.52

Scanner-specific classification rates are available in Supplementary Table S2.

Abbreviations: F, female; HP, healthy participants; M, male; PD, Parkinson’s disease.

Overall, the results highlight that site classification using these MRI observational data is generally possible. As can be seen in Table 2, models trained with raw T1-weighted and intensity-harmonized T1-weighted MRI scans performed similarly in terms of accuracy (85%). Classification rates for biological and non-biological variables are also highly similar. For instance, averaged sex, age, disease status, and scanner rates achieved accuracies of 86%, 84%, 85%, and 87%, respectively. These results suggest that a simple intensity correction is not sufficient for removing site information.

Although the model’s results employing the log-Jacobian maps were considerably lower in accuracy (54%), the model could still identify sites with an accuracy substantially better than the chance level. These results suggest that even removing intensity information completely is insufficient to remove site-related information. Figure 3A supports this result, showing that the model trained using the log-Jacobians maps could identify 18 sites precisely.

Furthermore, Figure 3A shows that 5 specific sites (BIOCOG, HAMBURG, OASIS, PPMI_20, and UKBB) were classified with very high accuracy for models trained with raw T1-weighted, harmonized T1-weighted, and log-Jacobians maps. For the misclassified sites, 2 patterns were identified (Figure 3A). First, data from sites tended to be misclassified as other sites managed by the same study. For instance, some PPMI sites were misclassified as other PPMI sites, and SBK got misclassified as UOA, 2 sites from the same study. Lastly, sites tended to get misclassified as OASIS, which is one of the studies providing a large number of scans from a single site but multiple scanner types to this database.

As expected, Figure 3B demonstrates that all models trained using the simulated data distribution (less biased) performed poorly on the site classification task, achieving accuracy at the chance level of around 10%.

Statistical likelihood ratio tests were performed on the 3 models trained on the real data distribution. These tests revealed that disease status and scanner type contributed significantly (*P*-value < .05) to the accuracy of the 3 models. Furthermore, age also contributed significantly to the model trained using the log-Jacobians maps. Table S3 shows the likelihood ratio test results for the 3 models and all biological and non-biological variables.

**Figure 4.**
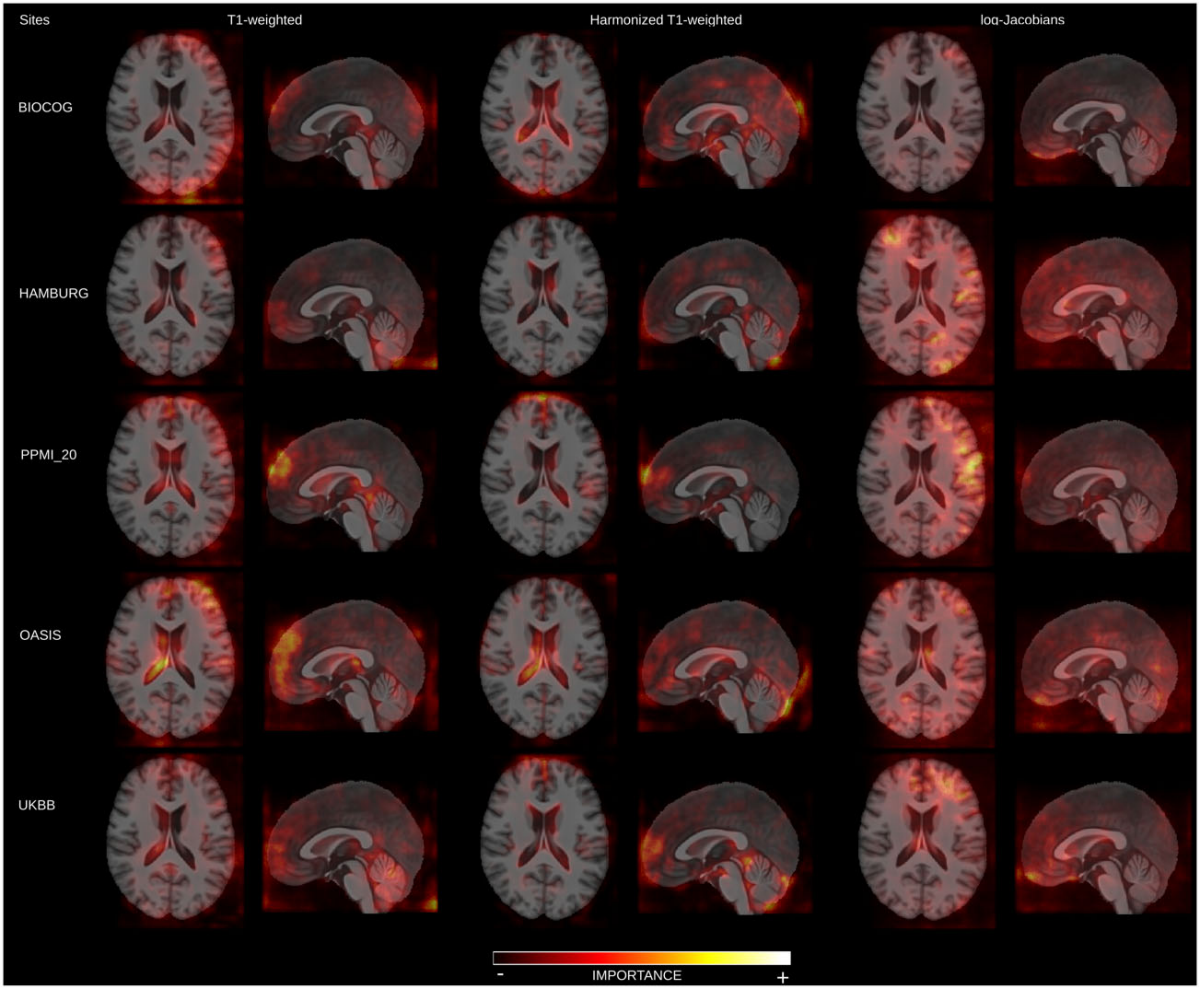
Saliency maps for 5 sites of the models trained in real data distribution.

Figure 4 highlights the brain regions used by the models to identify 5 sites, which achieved high accuracy: BIOCOG, HAMBURG, OASIS, PPMI_20, and UKBB. Figures S4–S6 present saliency maps for all sites correctly identified by each model for completeness purposes.

Overall, it can be observed that each model focused on distinct areas of the brain, suggesting that some more local and global artifacts and distortions are present in the data. Models trained with raw T1-weighted and harmonized T1-weighted MRI scans highlight more similar brain areas as most informative. However, it is noticeable that the frontal lobe and some background regions are less important in the intensity-harmonized T1-weighted MRI scans. Although our result suggests that harmonizing the intensities reduced the ability of the model to rely on areas of the brain that are more susceptible to artifacts caused by differences in the head coil and during image registration, originating sites are still distinguishable by the model. Concerning the log-Jacobian maps’ saliency maps, brain structures affected most by age are highlighted as important, such as in the ventricles and sulci, supporting the statistical likelihood ratio test results that disease status, age, and scanner types contributed significantly (*P*-value < .05) to this model. Nevertheless, it is essential to note that those brain areas correspond to regions that are usually more challenging for registration methods to align. While it is very complicated to disentangle the contribution of the biological age and possible registration errors, our results suggest that even when the data contain mainly morphological information, site classification is still possible.

## Discussion

The main finding of this work is that machine learning models can classify sites directly from multisite MRI data that were initially compiled for a disease classification task, even after intensity harmonization or complete removal of intensity information. This finding suggests that there is site-specific information in brain MRI scans that cannot be removed by using rather simple intensity harmonization techniques used as standard preprocessing steps prior to model training. Moreover, log-Jacobian maps, which mostly contain morphological information, are also not capable of completely removing site-specific information, which could, for example, be caused by MRI magnetic field homogeneities or other imaging artifacts. Although this work specifically investigated the ability of DL to perform site classification, we believe that the features used by this model after intensity harmonization could also be used as a shortcut(s) by a disease classifier trained on the same data. This has many practical implications regarding the training of machine learning models based on multisite data and suggests that more advanced data harmonization techniques may be required but also that trained models need to be carefully evaluated to exclude the chance that shortcuts were used in the background. Overall, these results may explain why many DL models do not generalize well or even fail when applied to new datasets that were acquired in centers that did not contribute to the training set.

Our analysis shows that disease status and scanner type significantly contributed to all models. Such biological and non-biological influences were also observed in previous studies. For example, Stanley et al.^8^ noticed a contribution of the pubertal score when classifying sex using a DL model. While Tardif et al.^9^^,^^10^ reported a strong influence of scanners, the magnetic field strength, and the imaging protocol in their voxel-based morphometry analysis, Nielson et al.^36^ observed similar influences in a DL model. Within this context, it is important to note that there are considerable differences in the patient distribution between some of the studies included in this work. For example, Biocog is a clinical study that aimed at examining representative patients across the whole disease spectrum (with the usual biases of patients who agree to be part of studies), while the Parkinson’s Progression Markers Initiative (PPMI) was primarily designed to investigate *de novo* patients with PD, leading to differences in disease stage as a potential bias that can be used as a shortcut. Moreover, it is important to note that the imbalanced number of subjects scanned using the same scanner type and protocol may add potential biases that could be used as shortcuts. In contrast to other well-known databases, such as the Alzheimer’s Disease Neuroimaging Initiative (ADNI) database, PPMI does not acquire and collect data using a harmonized imaging protocol. Instead, the data available in PPMI were mostly acquired using local imaging protocols that can differ considerably between sites. In a real-world scenario, it is unlikely that every medical center will have access to the same scanner device, which justifies treating each center managed by PPMI as an individual site in our work and highlights the importance of our work analyzing this unique, realistic database.

Our results clearly show that simple intensity normalization,^31^ the most used data harmonization technique for training DL models, is not optimal and cannot remove all site-related information from raw T1-weighted MRI scans. Although histogram matching may make the intensity distribution more similar between sites, it is not able to correct for local intensity differences, for example, caused by coil inhomogeneities or local/global artifacts and distortions that may be scanner and protocol dependent. Similar results were observed by Glocker et al.^13^ when analyzing scanner effects between 2 sites. The results of the present study expand those findings and show that this is even true when using databases that were collected from a much larger number of centers. Thus, the general assumption that using data from many centers helps to train more generalizable machine learning models may not hold true and needs to be carefully revisited.

Our findings indicate that log-Jacobian maps, which completely remove intensity information, do not fully eliminate site-related information. A potential reason for this may be that the model trained with these maps may rely on brain areas that are traditionally challenging for registration methods to align accurately. While disentangling the contribution of brain morphology from potential registration errors is complex, our results suggest that site classification remains possible, even when the data predominantly consists of morphological information.

Overall, our findings indicate that various factors within cohorts, such as disease duration, sex, and age distribution of patients, the number of subjects scanned at each site, the representation of disease status (PD vs healthy) at each site, and the heterogeneity of scanner devices across sites, are potential features that can be exploited by DL models to accurately discern originating sites. Thus, these findings highlight the importance of and need for more research on advanced data harmonization and bias mitigation techniques.

Recently, more complex data harmonization strategies that utilize (generative) DL techniques such as generative adversarial networks (GANs) have been explored. For example, Dewey et al.^37^ proposed a DL model that generates image data with consistent contrast, whereas Dinsdale et al.^11^ employed 2 networks in an adversarial training technique—one to remove protocol-specific effects and the other to predict brain aging. More generally, the objective of GANs is to generate images that preserve structural differences while altering their appearance. For instance, the image-to-image translation technique can translate MRI into computed tomography (CT) datasets.^38^ On the other hand, the style transfer technique applies a reference image’s style, such as the intensities of an atlas, to the structural information of the datasets of interest.^39^ While these advanced approaches hold promise for improved results compared to the simple histogram matching used in our study, it is essential to consider that preserving structural information may still retain local and global distortions as in the case of log-Jacobian maps, potentially leading to shortcut learning opportunities. Thus, it may be important to consider additional methods during or after imaging, such as distortion correction.^40^

It is essential to highlight some of the limitations of this work. First, although we demonstrated that site classification is possible and identified potential shortcuts, we did not show that the identified factors are indeed used as shortcut learning, which will be explored in more detail in future work. Second, our statistical analysis using logistic regression assumes that the effect of biological and non-biological variables on the accuracy of the models is linear. A non-linear analysis may be necessary to investigate if sex and age are indeed insignificant. Third, although saliency maps are widely used to interpret machine learning models’ decisions, it is known that gradient-based attribution maps are not always perfect and may highlight some background regions close to the regions of high informative value, even when they are null (eg, all black or zeros). Lastly, we only analyzed the SFCN model in this work. Thus, results could be different for other machine learning models.

## Conclusion

This work investigated the ability of machine learning models to classify sites from raw T1-weighted MRI scans with and without applying intensity harmonization techniques. To the best of our knowledge, this is the first work that implemented site classifiers using a very large multisite MRI database collected from across 41 sites. Our results demonstrate that MRI scans encode relevant site-specific information that models could use as shortcuts that cannot be removed using simple intensity harmonization methods. Thus, caution is advised when developing machine learning applications, especially when using multisite datasets, as biological and non-biological variables may introduce biases into the model. Most importantly, caution is essential when drawing conclusions from machine learning models trained in a multisite setup as a disease classifier (modeled task) could end up being a secret site classifier (shortcut/biases task) due to complex confounding factors encoded in the data.

## Supplementary Material

ocad171_Supplementary_Data

## Data Availability

Image data used were provided, in part, by the OASIS-3 project (Principal Investigators: T. Benzinger, D. Marcus, J. Morris; NIH P50 AG00561, P30 NS09857781, P01 AG026276, P01 AG003991, R01 AG043434, UL1 TR000448, R01 EB009352), by the PPMI-a, public–private partnership funded by Michael J. Fox Foundation, by the OpenfMRI database (accession number ds000245), and by the UK Biobank (application number 77508).
