# Supplementary material for: Image-encoded biological and non-biological variables may be used as shortcuts in deep learning models trained on multisite neuroimaging data
Source: J Am Med Inform Assoc. 2023 Sep 5;30(12):1925–33. doi: 10.1093/jamia/ocad171 (PMC10654841; doi:10.1093/jamia/ocad171)
Supplement: ocad171_Supplementary_Data [file ocad171_supplementary_data.docx]

**Image-encoded biological and non-biological variables may be used as shortcuts in deep learning models trained on multi-site neuroimaging data**

**Supplementary Material**

Table 1: Site-specific data distribution. M=male, F=female.

| Disease status | Parkinson’s disease | | Healthy participants | |
| --- | --- | --- | --- | --- |
| Sites | Sex (M / F) | Age (<60 / 60+) | Sex (M / F) | Age (<60 / 60+) |
| BIOCOG | 25 / 20 | 0 / 45 | 28 / 21 | 0 / 49 |
| C-BIG | 36 / 30 | 16 / 50 | 1 / 9 | 3 / 7 |
| HAMBURG | 52 / 22 | 23 / 51 | 0 / 0 | 0 / 0 |
| HMC | 1 / 2 | 0 / 3 | 0 / 0 | 0 / 0 |
| Japan_dataset | 13 / 17 | 4 / 26 | 7 / 8 | 4 / 11 |
| JGH | 2 / 0 | 0 / 2 | 0 / 0 | 0 / 0 |
| MUC | 7 / 3 | 3 / 7 | 0 / 0 | 0 / 0 |
| Neurocon | 16 / 10 | 4 /22 | 4 / 12 | 6 / 10 |
| OASIS | 0 / 0 | 0 / 0 | 252 / 151 | 75 / 328 |
| PD_MCI_CALGARY | 53 / 26 | 0 / 79 | 20 / 22 | 0 / 42 |
| PD_MCI_PLS | 26 / 15 | 15 / 26 | 10 /11 | 7 / 14 |
| PPMI_10 | 9 / 7 | 6 / 10 | 4 / 3 | 3 / 4 |
| PPMI_12 | 10 / 9 | 5 / 14 | 5 / 5 | 4 / 6 |
| PPMI_13 | 17 / 5 | 10 / 12 | 2 / 3 | 0 / 5 |
| PPMI_14 | 2 / 0 | 1 / 1 | 0 / 0 | 0 / 0 |
| PPMI_15 | 11 / 5 | 6 / 10 | 5 / 5 | 4 / 6 |
| PPMI_16 | 12 / 7 | 8 / 11 | 4 / 4 | 4 / 4 |
| PPMI_17 | 8 / 3 | 5 /6 | 6 / 3 | 5 / 4 |
| PPMI_18 | 7 / 6 | 2 / 11 | 4 / 0 | 2 / 2 |
| PPMI_19 | 15 / 7 | 11 / 11 | 8 / 4 | 9 / 3 |
| PPMI_20 | 20 / 17 | 22 / 35 | 6 / 7 | 7 / 6 |
| PPMI_21 | 11 / 3 | 9 / 5 | 0 / 0 | 0 / 0 |
| PPMI_22 | 14 / 4 | 5 / 13 | 10 / 2 | 3 / 9 |
| PPMI_23 | 3 / 9 | 6 / 6 | 9 / 3 | 4 / 8 |
| PPMI_25 | 14 / 5 | 7 / 12 | 7 / 2 | 5 / 4 |
| PPMI_26 | 8 / 6 | 5 / 9 | 1 / 0 | 0 / 1 |
| PPMI_27 | 12 / 9 | 8 / 13 | 8 / 3 | 6 / 5 |
| PPMI_28 | 13 / 7 | 9 / 11 | 4 / 1 | 2 / 3 |
| PPMI_29 | 4 / 7 | 2 / 9 | 6 / 0 | 3 / 3 |
| PPMI_30 | 2 / 1 | 1 / 2 | 2 / 0 | 0 / 0 |
| PPMI_51 | 11 / 7 | 5 / 13 | 5 / 2 | 4 / 3 |
| PPMI_52 | 15 / 8 | 8 / 15 | 4 / 7 | 3 / 8 |
| PPMI_53 | 2 / 3 | 3 / 2 | 3 / 4 | 6 / 1 |
| PPMI_55 | 3 / 0 | 1 / 2 | 1 / 0 | 0 / 1 |
| PPMI_59 | 5 / 1 | 2 / 4 | 0 / 0 | 0 / 0 |
| RUH | 3 / 3 | 0 / 6 | 0 / 0 | 0 / 0 |
| SALD | 0 / 0 | 0 / 0 | 78 / 0 | 34 / 44 |
| SBK | 3 / 0 | 0 / 3 | 0 / 0 | 0 / 0 |
| Taowu | 8 / 9 | 1 / 16 | 12 / 8 | 3 / 17 |
| UKBB | 28 / 20 | 4 / 44 | 119 / 78 | 37 / 160 |
| UOA | 21 / 12 | 5 / 28 | 0 / 0 | 0 / 0 |

Table 2: Classifiers classification rates based on scanner types for real data distribution

| Scanner type / Classifier | T1-weighted | Harmonized T1-weighted | Jacobians |
| --- | --- | --- | --- |
| GE Discovery 750 | 0.90 | 0.96 | 0.53 |
| GE Genesis Signa | 1.0 | 1.0 | 0.50 |
| GE Optima MR450 | 1.0 | 1.0 | 0.50 |
| GE Signa Excite | 1.0 | 1.0 | 1.0 |
| GE Signa Hdxt | 0.81 | 0.87 | 0.56 |
| Philips Achieva | 1.0 | 0.90 | 0.27 |
| Philips Gyroscan NT | 1.0 | 0.66 | 0.0 |
| Philips Intera | 0.0 | 0.0 | 0.0 |
| Siemens Avanto | 0.85 | 0.85 | 0.42 |
| Siemens Biograph_mMR | 0.90 | 0.70 | 0.80 |
| Siemens Espree | 0.54 | 0.54 | 0.36 |
| Siemens Prisma | 0.75 | 0.87 | 0.25 |
| Siemens Prisma_fit | 0.83 | 0.83 | 0.05 |
| Siemens Skyra | 0.97 | 0.98 | 0.74 |
| Siemens Sonata | 1.0 | 1.0 | 1.0 |
| Siemens Symphony | 1.0 | 1.0 | 0.0 |
| Siemens Trio | 0.91 | 0.91 | 0.43 |
| Siemens Trio Tim | 0.77 | 0.77 | 0.54 |
| Siemens Verio | 0.75 | 0.75 | 0.33 |

Table 3: Likelihood ratio test ratio results show the contribution of each biological and non-biological variable to the accuracy of the models trained on the real data distribution (* p-value < 0.05). The likelihood ratio test ratio is a metric utilized to evaluate the relative adequacy of two nested statistical models. It determines whether one model significantly outperforms the other in explaining the observed data.

| Data distribution | Input | Age | Sex | Characteristic (PD vs HP) | Scanner models |
| --- | --- | --- | --- | --- | --- |
| Real | T1-weighted | 0.26 | 0.15 | 1.56e-08* | 6.60e-05* |
|  | Harmonized T1-weighted | 0.65 | 0.07 | 1.22e-09* | 3.73e-06* |
|  | log-Jacobians | 0.0002* | 0.06 | 2.99e-16* | 2.91e-09* |





Fig. 1: Disease status distribution per site





Fig. 2: Sex distribution per site





Fig.3: Age distribution per site


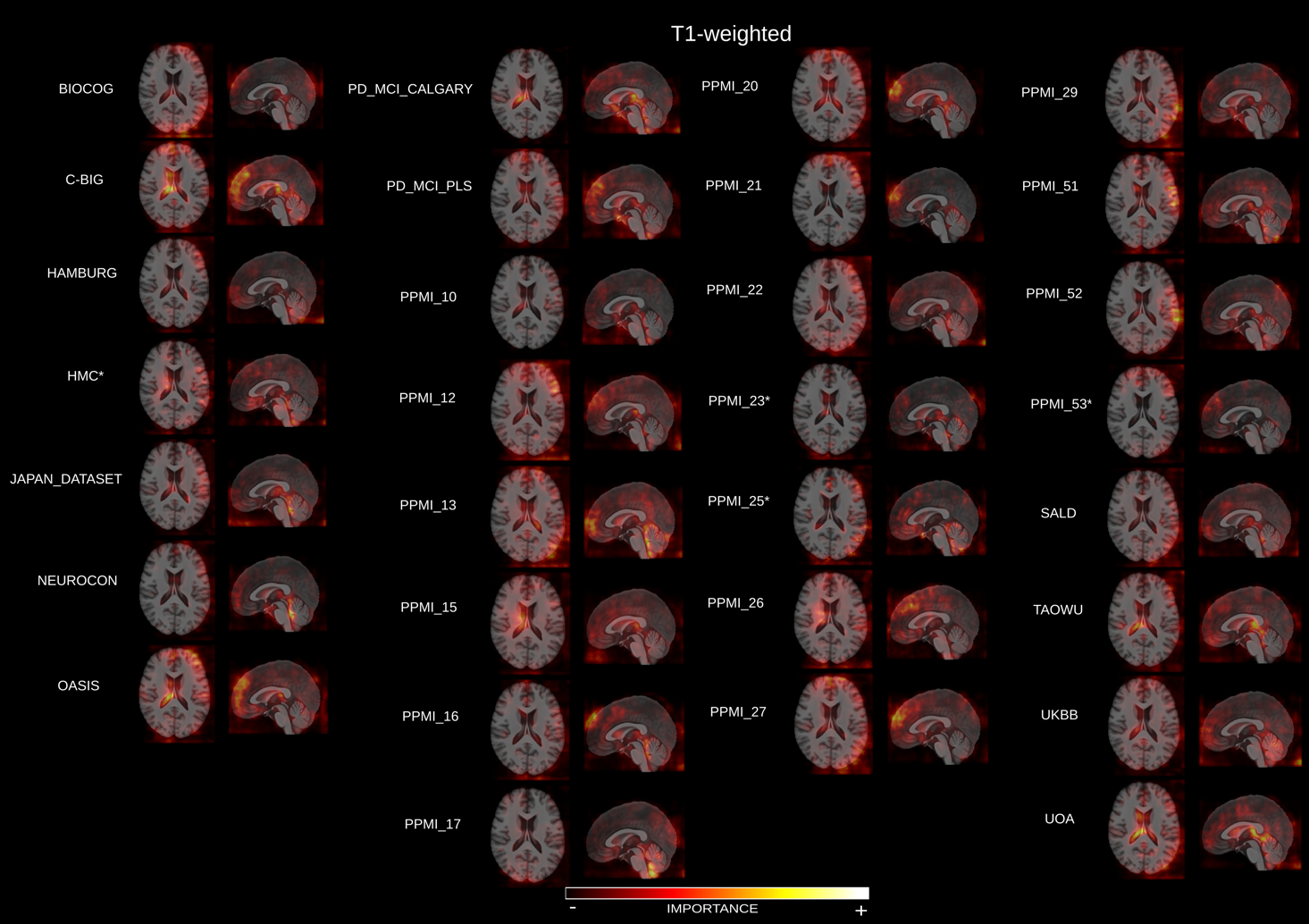
Fig. 4: Saliency maps from the model trained using T1-weighted


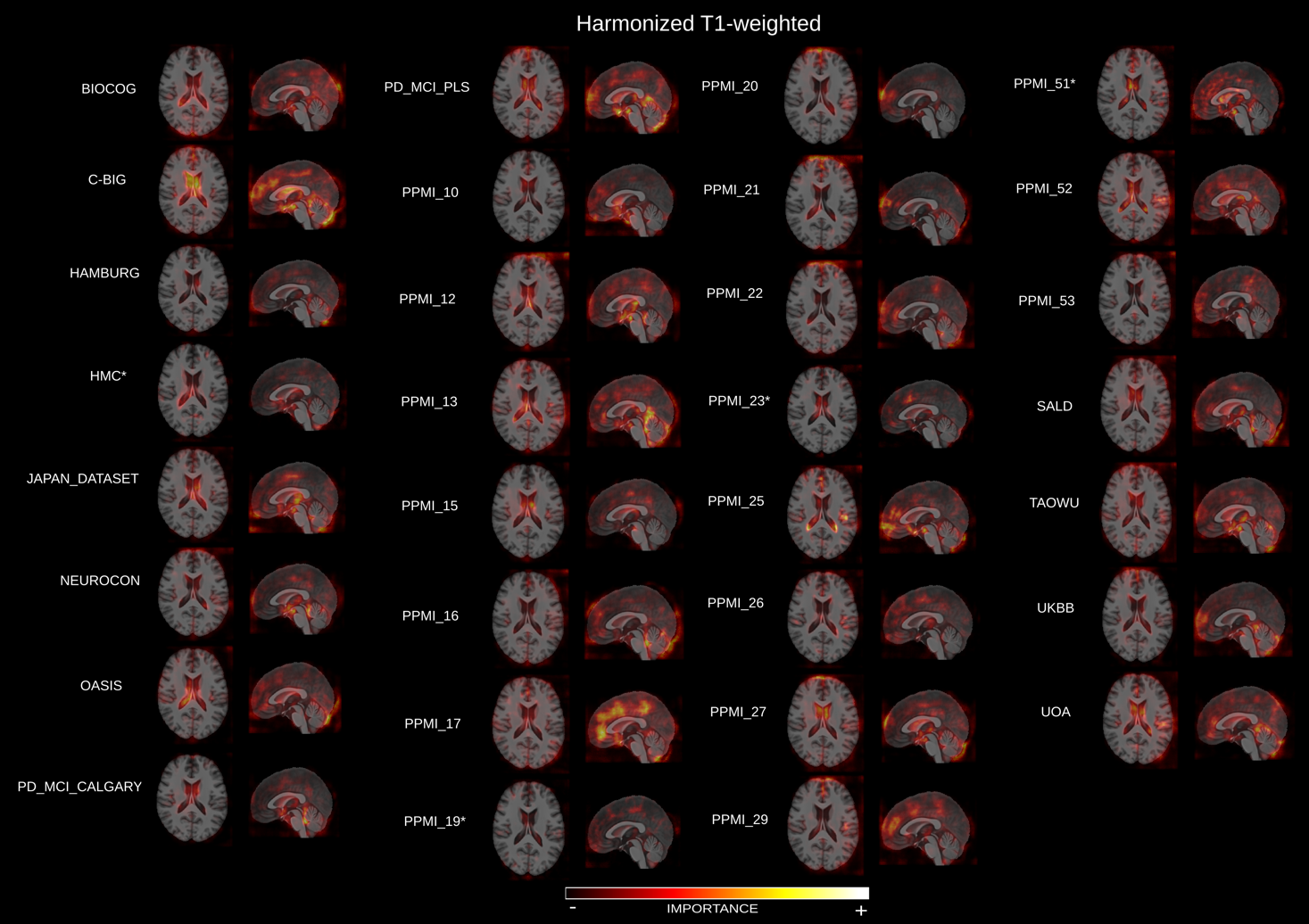
Fig. 5: Saliency maps from the model trained using harmonized T1-weighted


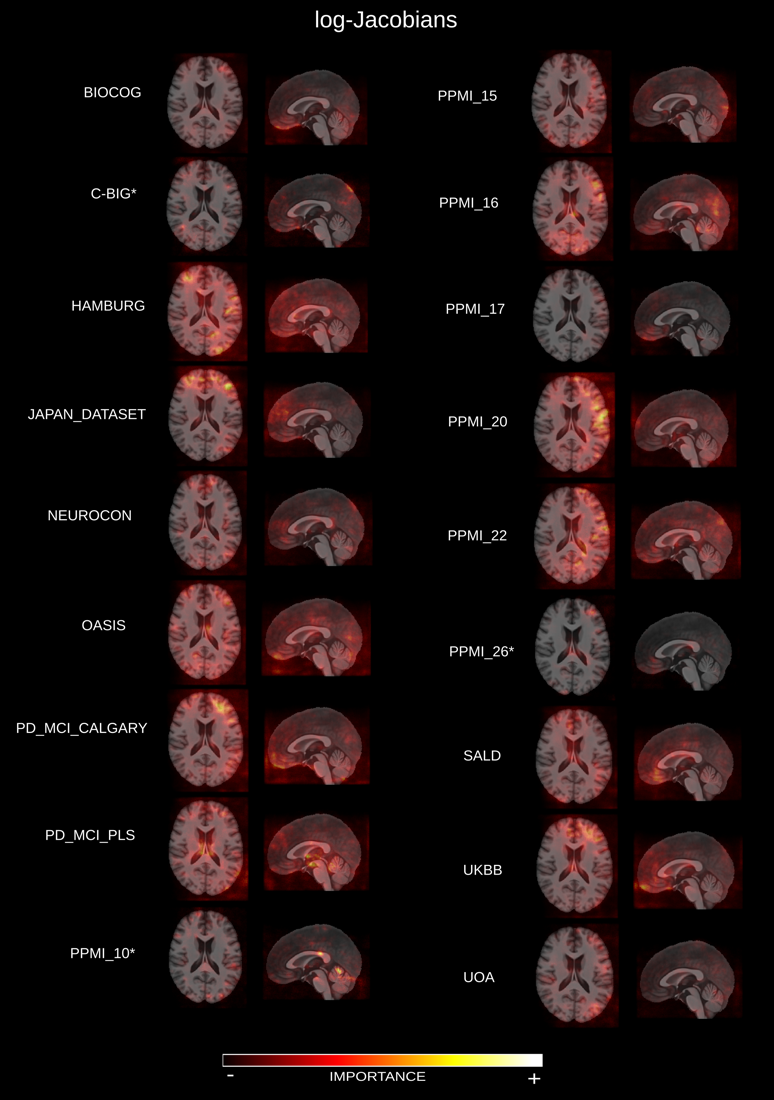


Fig. 6: Saliency maps from the model trained using log-Jacobians
